# CircRNA ACVR2A Sponges miR-1290 to Modulate Cell Progression in Gastric Cancer

**DOI:** 10.1155/2022/9461054

**Published:** 2022-02-09

**Authors:** YingYing Zhuang, LiYa Li, HaiNing Wu, TaiYong Fang

**Affiliations:** ^1^Department of Gastroenterology, The Second Affiliated Hospital of Fujian Medical University, Quanzhou, China; ^2^Department of Ultrasound, The Second Affiliated Hospital of Fujian Medical University, Quanzhou, China; ^3^Department of Neurology, The Second Affiliated Hospital of Fujian Medical University, Quanzhou, China

## Abstract

**Background:**

In recent years, the abnormal expression of circRNAs has been identified to be strongly associated with tumor tissues. In this study, we focused on circACVR2A with a remarkably upregulated expression in gastric tissues and further explored its role in the pathogenic progression of gastric cancer (GC).

**Methods:**

The differentially expressed circACVR2A in GC tissues and four cell lines (MKN-45, SNU-1, HGC-27, and SGC-7901) was identified by qRT-PCR method. Then, the effect of circACVR2A and miR-1290 on HGC-27 cell proliferation was measured by CCK8 and the colony formation methods. The effect of circACVR2A and miR-1290 on HGC-27 cell metastasis was estimated by transwell assay. The interaction of circACVR2A and miR-1290 was further detected.

**Results:**

The relative level of circACVR2A in GC tissues and cell lines is remarkably upregulated. The downregulation of circACVR2A promotes GC cell proliferation and metastasis and suppressed the expression level of E-cadherin and Vimentin. The miR-1290 inhibitor reversed the effect of circACVR2A on cell progression in GC cell.

**Conclusion:**

circACVR2A competitively sponged miR-1290 and was exerted as a tumor suppressor gene oncogene via a circACVR2A/miR-1290 axis, suggesting it as a possible biomarker for GC therapy.

## 1. Introduction

Gastric cancer is the most common type of diagnosed cancer around the world, with a high mortality rate, accounting for 8.2% of all cancer deaths [1]. Compared with other parts of the world, East Asia is a high incidence area of GC, with a significant increase in incidence rate 43% of gastric cancer patients are in China [2,3]. GC has been considered to be a progressive process starting from chronic persistent inflammatory response, and infection has a high tendency of tumor invasion and metastasis [4,5]. Most patients with early gastric cancer have no symptoms and are difficult to diagnose. When anorexia, dyspepsia, weight loss, and abnormal pain symptoms occur, they are usually in the late stage, and the survival rate is lower than 30% [6]. At present, the treatment methods for GC include surgical resection, chemotherapy, and targeted therapy [7]. Although the diagnosis and treatment strategies of GC have been improved, it is still a big challenge to completely remove the tumor through surgery, radiotherapy, and chemotherapy. The prognosis of patients with GC is common poor. Therefore, there is an urgent need to find out the underlying mechanism of GC progression. It is necessary to identify effective diagnostic biomarkers and treatment strategies, which is essential to promote the survival rate of GC patients.

Circular RNAs (circRNAs) are a type of endogenous noncoding RNA molecules characterized by closed covalent rings, which do not contain 5′-terminal cap structure and 3′ -terminal poly-A tail. They are widely distributed in various eukaryotes [8,9]. Compared with their homologous linear RNAs, circular RNAs are resistant to RNA because of their structural characteristics of forming closed covalent rings and showed better stability [10–12]. Studies have shown that cyclic RNAs not only are byproducts of splicing, but also have rich biological functions. They can modulate gene expression at the transcriptional or posttranscriptional level via interacting with microRNAs or binding with other molecules [13]. MicroRNAs can interact with microRNAs through their own microRNA binding sites. They can bind to microRNA efficiently and adsorb microRNA like sponge and then affect the effect of microRNA on its target. Circular RNA plays a crucial function in the occurrence and development of a variety of cancers and chronic diseases, including gastric cancer [14,15].

GC is a common malignant tumor of digestive tract, and many long noncoding RNAs have been reported to be strongly associated with GC progression [16,17]. More and more studies have found that there are differences in the expression of many circular RNAs in GC tissues and the corresponding adjacent normal tissues, but the change trend of the differential expression of circular RNA in GC tissues is different, both upregulated and downregulated, and the relationship with clinical characteristics is not the same. Some studies have shown that circPVT1 is highly expressed in GC. By binding with mir-125, circPVT1 inhibits the binding of mir-25 with target gene E2F2 and upregulates the expression level of E2F2, thus promoting cell proliferation [18]. CircLARP4 is downregulated in gastric cancer tissues and is related to the pathological stage, which directly affects the survival rate of gastric cancer patients after diagnosis. CircLARP4 can inhibit the expression of LATS1 by inhibiting the effect of miR-424, thus inhibiting the proliferation and invasion in GC cells [19]. However, there are few studies on molecular mechanism of circRNA in GC, and the upstream and downstream signaling networks are elusive. Therefore, more in-depth studies are still needed to clarify the detailed mechanism and the relative signaling pathways.

In the current study, we revealed that circACVR2A was upregulated in GC tissues and four cell lines. The circACVR2A could influence GC cell proliferation and metastasis via interacting with miR-1290 as a miRNA sponge. These findings provide a new insight into the GC progression and are exerted as a potential biomarker for prognostic of GC.

## 2. Materials and Methods

### 2.1. Clinical Samples

GC tissues (*n* = 20) and their matched adjacent normal epithelial tissues (*n* = 20) have been obtained from the Second Affiliated Hospital of Fujian Medical University. All these clinical samples have been immediately frozen in liquid nitrogen after surgical removal and stored at −80°C for further studies. The application of human GC clinical samples has been examined and approved by the Ethical Committee of The Second Affiliated Hospital of Fujian Medical University. All patients has agreed and written the informed consent.

### 2.2. Cell Culture and Cell Transfection

A normal human cell GES-1 and four human GC cell lines, MKN-45, SNU-1, SGC-7901, and HGC-27, have been acquired from American Type Culture Collection (ATCC). All these cells were seeded in the culture plate under 37°C constant temperature with 5% CO2 humidified atmosphere. These cells were cultured in the DMEM containing 10% FBS and the penicillin-streptomycin mixed solution. According to instructions of Lipofectamine 2000 transfection kit (Invitrogen, Carlsbad, CA, USA), the prepared cells were then transfected with 50 nM si-circACVR2A or si-NC. After 48 hours, the relative level of circACVR2A was examined by qRT-PCR. The siRNAs targets to circACVR2A in this study were displayed:

  si-circACVR2A#1: 5′-AACTCAAGTGCTATACTTGGTTT-3′
  si-circACVR2A#2: 5′-ACTTGTTCCAACTCAAGTGCTTT-3′
  si-circACVR2A#3: 5′- ACTCAAGTGCTATACTTGGTATT-3′

### 2.3. RNA Isolation, Reverse Transcription, and qRT-PCR

The RNA was isolated from GC tissues and cell lines by TRIzol (Invitrogen, Carlsbad, USA). Prepared RNA was then quantified by the Nanodrop spectrophotometer (Thermo, DE, USA). 500 ng of the prepared RNA was reversely transcribed into the cDNA. The qPCR assay was performed. All the primer sequences utilized here were acquired. GAPDH and U6 have been utilized to be the internal controls for circRNA and miRNA, respectively. The amplification reaction was set up as normal process. All the experiments in triple independent examination have been assayed as average. The relative level has been assayed via the comparative 2^−ΔΔCT^ assay.

### 2.4. CCK8 Assay

HGC-27 cell proliferation was next examined. HGC-27 cells were cultured in a 96-well plate with 1 × 10^5^ cells per well and further incubated for 24, 48, and 72 hours in the incubator. After a certain time culture, the CCK8 reagent with 10 ul was added to the well for additional 1 hour. And then, HGC-27 cell proliferation was examined by microplate reader (Bio-Rad, USA) with an absorbance wavelength of 450 nm. All the experiments in triple independent examination have been assayed as average.

### 2.5. The Colony Formation Assay

HGC-27 cells were firstly cultured in a 6-well plate with 100 cells/well. After transfection, the DMEM medium was discarded, and PBS buffer was injected into the cells. Then, the crystal violet with 1 ml (0.1%; Sigma-Aldrich, MO, USA) was injected for staining. After 1-hour incubation, the colony size with more than 50 um has been finally examined. All the experiments in triple independent examination have been assayed as average.

### 2.6. Transwell Assay

The polycarbonate membrane Boyden chamber (Corning, Kennebunk, USA) has been utilized for migration and invasive ability. According to the protocol, transfected HGC-27 cells were resuspended in medium, and an 8 um pore chamber with/without 25 mg Matrigel was placed into the well (BD Biosciences, CA, USA). The 10% FBS-DMEM medium was injected into the lower chamber. After 24-hour incubation, HGC-27 cells could migrate to the lower chamber. The rate of migration and invasion has been finally calculated from counting in at least five random fields per membrane.

### 2.7. Luciferase Activity Assay

The luciferase activity assay has been carried out to determine whether miR-1290 binds to the circACVR2A 3′-UTR. The circACVR2A 3′-UTR (circACVR2A-WT) and its mutant (circACVR2A-MUT) have been commercially purchased and constructed into the psiCHECKTM2 vector. HGC-27 cells were cultured in a 24-well plate with 2 × 10^5^ cells/well. When they grew to 80% confluence, the reporter plasmid circACVR2A-WT/circACVR2A-MUT and miR-1290 mimics were transfected with HGC-27 by Lipofectamine 2000. After transfection, the relative luciferase activity was examined.

### 2.8. Protein Extraction

HGC-27 cells were transfected with si-circACVR2A or LV-circACVR2A or miR-1290 inhibitor. After 48-hour incubation, the cells were harvested and resuspended in the lysis buffer with 1 mM PMSF. After centrifugation, the total protein was extracted for HGC-27 and directly quantified by the Bradford kit (Bio-Rad).

### 2.9. Immunoblot Analysis

The prepared protein with 10 ug from each sample was denatured and then injected into a 12% SDS-PAGE. Subsequently, the proteins were transferred to the PVDF membrane, which was further blocked with 10% milk and incubated with the primary antibodies for 24 hours at 4°C. In another day, the treated membrane was further incubated with the mouse/rabbit antibodies and finally examined by the ECL-plus reagents. The protein band was detected and quantified via Quantity one software (Bio-Rad). All the experiments were carried out in triple independent assays. The E-cadherin antibody (sc-21791) and Vimentin antibody (sc-66002) were acquired from Santa Cruz Biotechnology (Dallas, USA). These antibodies were diluted at 1:1000, and the actin antibody was diluted at 1:5000.

### 2.10. miRNA Pull-Down Assay

Biotin miRNA-1290 probe was labeled by in vitro transcription, and then the total RNA extract of cells overexpressing CirRNA ACVR2A was incubated to form a complex. The complex can be combined with streptavidin labeled magnetic beads to separate from other components in the incubation solution. After the complex was eluted, whether CirRNA ACVR2A interacted with RNA was detected by qPCR.

### 2.11. Statistical Analysis

Data was exhibited as means and standard deviations (SD) from triple independent assays. The statistical significance has been evaluated by Student's *t*-test in Graphpad Prism 6.0 (SanDiego, USA). The data has been considered significant at a *p* value (<0.05).

## 3. Results

### 3.1. Aberrant Upregulation of circACVR2A in GC Tissues and Cell Lines

**Figure 1:**
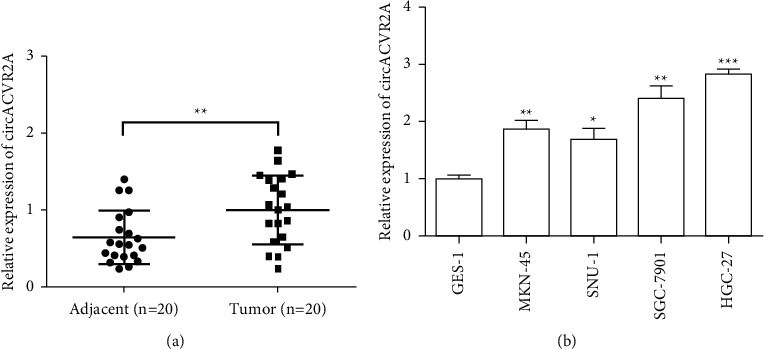
CircACVR2A is upregulated in GC tissues and cell lines. (a) The relative expression of circACVR2A in 20 pairs of GC tissues and its adjacent tissues examined by qRT-PCR. (b) The relative expression of circACVR2A in GES-1, MKN-45, SNU-1, SGC-7901, and HGC-27 cells examined by qRT-PCR. The data is exhibited as the mean ± SD. ^*∗*^*p* < 0.05, ^*∗∗*^*p* < 0.01, and ^*∗∗∗*^*p* < 0.001.

The relative level of circACVR2A in GC tissues (*n* = 20) and cell lines was estimated. circACVR2A exhibited a significant upregulation in contrast to the adjacent tissues (*n* = 20; *p* < 0.01; Figure 1(a)). The similar findings have been observed from the GC cell lines (MKN-45, SNU-1, SGC-7901, and HGC-27) in contrast to the normal gastric epithelial cell (GES-1; Figure 1(b)). As compared with GES-1, circACVR2A was upregulated 1.93-fold on average in MKN-45 (*p* < 0.01), 1.78-fold on average in SNU-1 (*p* < 0.05), 2.45 on average in SGC-7901 (*p* < 0.01), and 2.67 on average in HGC-27 (*p* < 0.001). Thus, we selected HGC-27 cells for the following studies.

### 3.2. circACVR2A Influenced the Proliferation and Metastasis In Vitro

**Figure 2:**
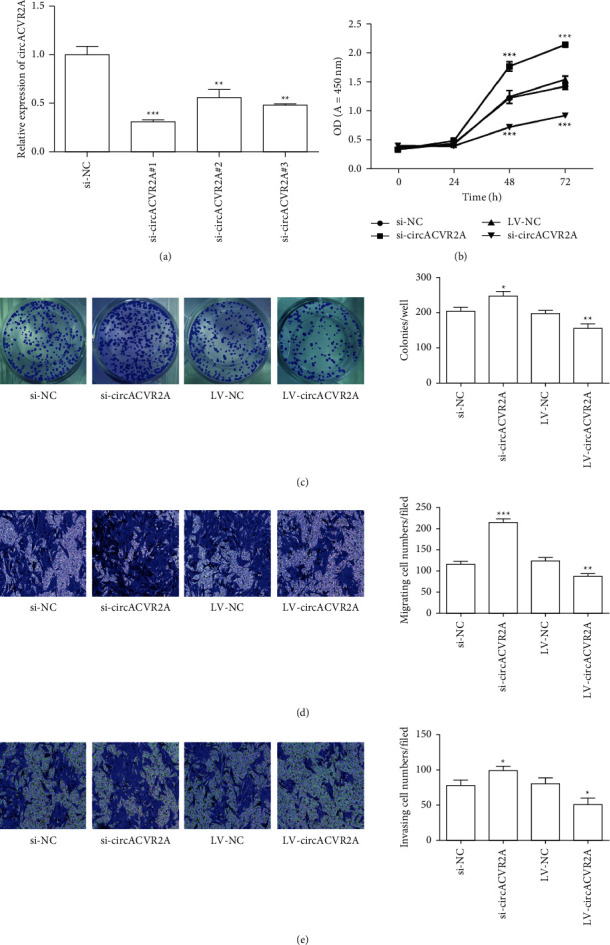
The effect of circACVR2A on GC cell proliferation and metastasis in HGC-27. (a) The relative expression of circACVR2A in HGC-27 cells transfected with three si-circACVR2A by qRT-PCR. (b) HGC-27 cell proliferation after circACVR2A downregulation or upregulation examined by CCK8 method. (c) HGC-27 cell colony formation after circACVR2A downregulation or upregulation detected by the colony formation assay. (d) and (e) HGC-27 cell migration and invasion were examined by transwell assay. The data is exhibited as the mean ± SD. ^*∗*^*p* < 0.05, ^*∗∗*^*p* < 0.01, and ^*∗∗∗*^*p* < 0.001.

We examined the cellular influence of circACVR2A in GC. At first, we synthesized the small interfering RNA (siRNA) interacting with the junction site of circACVR2A to downregulate circACVR2A expression level in HGC-27 cells. Obviously, these three siRNAs significantly downregulated the circACVR2A expression level (Figure 2(a)). In contrast to NC group, si-circACVR2A#1 was downregulated 0.33-fold on average (*p* < 0.001); si-circACVR2A#2 was downregulated 0.65-fold (*p* < 0.01); and si-circACVR2A#3 was downregulated 0.41-fold (*p* < 0.01). Thus, we utilized si-circACVR2A#1 in the following experiments due to its highest inhibitory efficiency. The CCK8 experiment indicated that downregulated circACVR2A remarkably promoted cell proliferation in HGC-27 cells at 48 and 72 hours in HGC-27 cells (*p* < 0.001) and upregulated circACVR2A remarkably suppressed the proliferation (*p* < 0.001; Figure 2(b)). The colony formation experiment exhibited that downregulated circACVR2A remarkably enhanced the proliferation (*p* < 0.01) and upregulated circACVR2A had a reverse effect on the formation of colony (*p* < 0.05; Figure 2(c)). Additionally, the ability of metastasis had also been estimated by transwell invasion experiment. Observed from Figures 2(d) and 2(e), the data showed that downregulated circACVR2A remarkably enhanced HGC-27 cell migration (*p* < 0.001) and invasive ability (*p* < 0.05), while upregulated circACVR2A remarkably inhibited HGC-27 cell migration (*p* < 0.01) and invasive ability (*p* < 0.05). The findings revealed that circACVR2A was strongly associated with the progression of HGC-27 cells.

**Figure 3:**
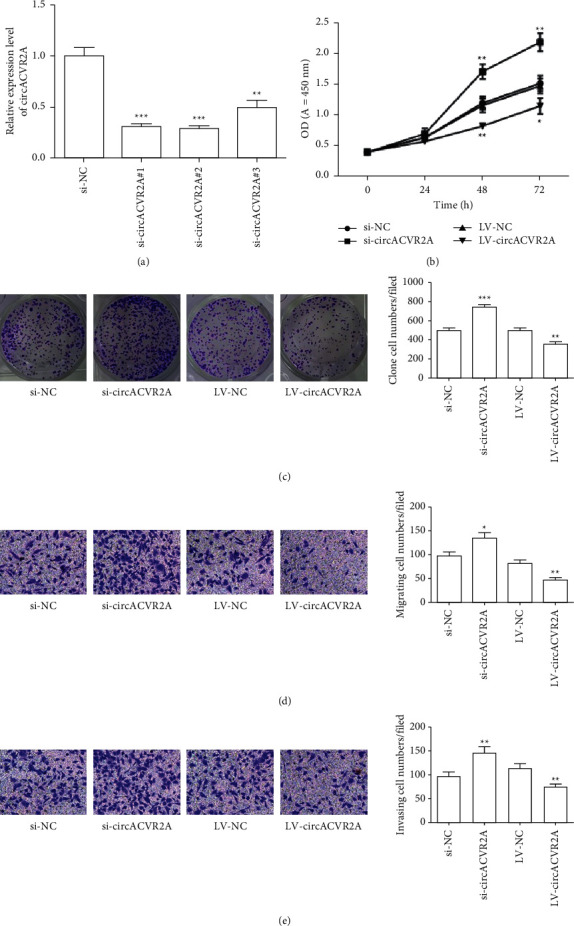
The effect of circACVR2A on GC cell proliferation and metastasis in MKN-45. (a) The relative expression in MKN-45 cell by qRT-PCR. (b) MKN-45 cell proliferation examined by CCK8 method. (c) MKN-45 cell colony formation detected by the colony formation assay. (d) and (e) MKN-45 cell migration and invasion were examined by transwell assay. The data is exhibited as the mean ± SD. ^*∗*^*p* < 0.05, ^*∗∗*^*p* < 0.01, and ^*∗∗∗*^*p* < 0.001.

We performed all the same experiments in MKN-45 cell. Results showed that all siRNAs significantly reduced the circACVR2A level (Figure 3(a)). The cell proliferation of MKN-45 cell after treatment was the same as in HGC-27 cell (Figure 3(b)). The colony formation experiment and transwell invasion experiment in MKN-45 cell revealed the similar trends as HGC-27 cell (Figures 3(c)–3(e)).

### 3.3. circACVR2A Affected Epithelial-Mesenchymal Transition (EMT) Signaling Pathway

**Figure 4:**
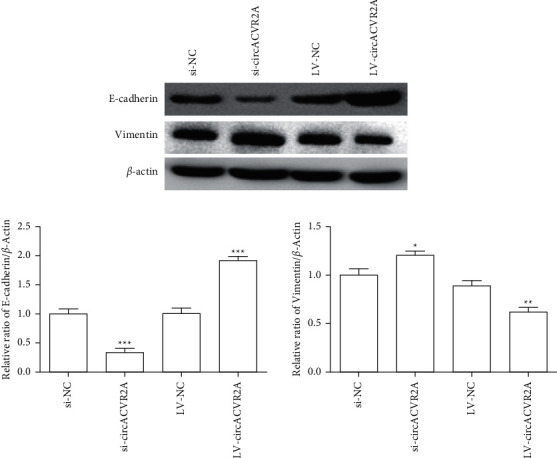
The effect of circACVR2A on EMT-related proteins. The protein expression of E-cadherin and Vimentin was examined after circACVR2A downregulation or upregulation in HGC-27 cells. The data exhibited as the mean ± SD. ^*∗*^*p* < 0.05, ^*∗∗*^*p* < 0.01, and ^*∗∗∗*^*p* < 0.001.

To further explore the underlying mechanism of circACVR2A, the EMT signaling pathway has been evaluated. As shown in Figure 4, downregulated circACVR2A led to a lower protein level of E-cadherin and a higher level of vimentin. Based on the above findings, it indicated that downregulated circACVR2A suppressed the proliferation and metastasis via directly downregulating the EMT pathway in GC cells.

### 3.4. circACVR2A Can Sponge miR-1290 in GC Cells

**Figure 5:**
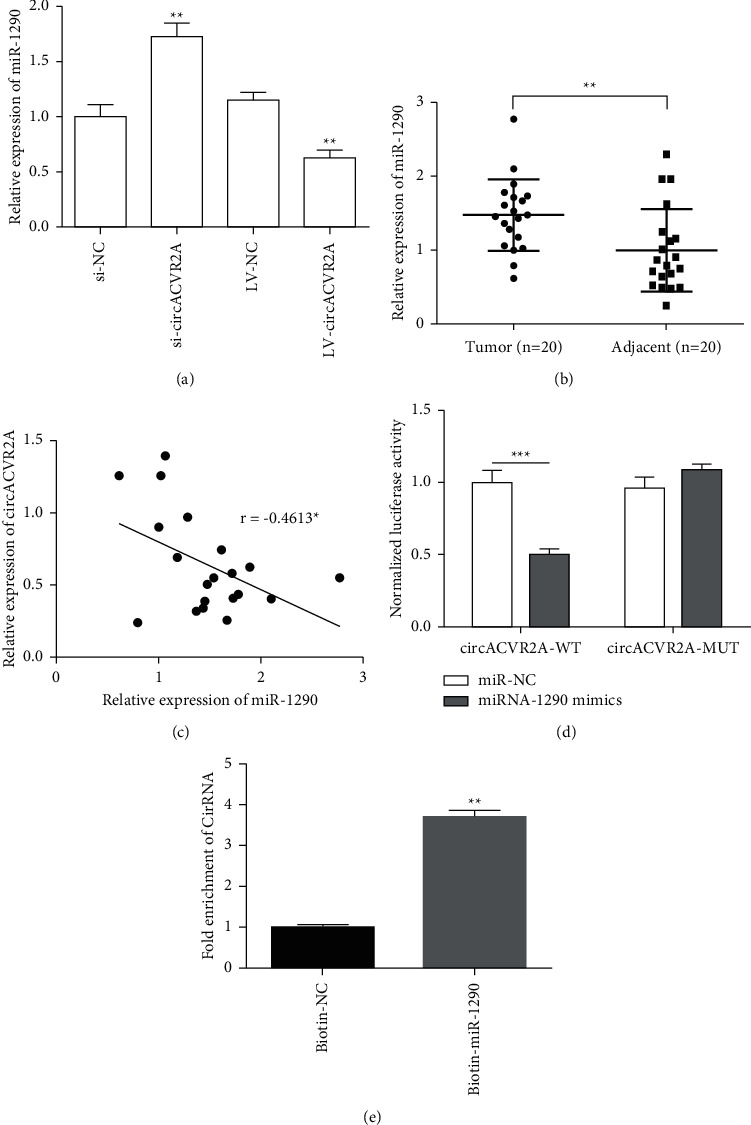
circACVR2A targeted miR-1290 to regulate its expression. (a) The relative expression of miR-1290 in HGC-27 cells after circACVR2A downregulation or upregulation examined by qRT-PCR. (b) The relative level of miR-1290 in GC tissues examined as compared to adjacent tissues. (c) The correlation expression between circACVR2A and miR-1290 in GC tissues. (d, e) The relationship between circACVR2A and miR-1290 in HGC-27 cells confirmed by the dual relative activity assay and miRNA pulldown assay. The data is exhibited as the mean ± SD. ^*∗*^*p* < 0.05, ^*∗∗*^*p* < 0.01, and ^*∗∗∗*^*p* < 0.001.

circRNAs are mainly positioned in the cytoplasm and bind to miRNA sponging [20,21]. Due to prediction of CircInteractome and miRanda, miR-1290 has been chosen to be a potential miRNA for the following studies. We designed a biotinylated circACVR2A probe and oligo probe to investigate whether circACVR2A can interact with miR-1290 in GC cells. As seen from Figure 5(a), the relative level of miR-1290 exhibited a negative association with circACVR2A expression. The relative level of miR-1290 showed a downregulated expression in GC tissues (*n* = 20) (*p* < 0.01; Figure 5(b)). The effect of circACVR2A on the relative level of miR-1290 was further assessed (Figure 5(c)). Downregulated circACVR2A significantly increased miR-1290 expression (*p* < 0.01) and upregulated circACVR2A remarkably decreased miR-1290 level (*p* < 0.01). The relative luciferase activity revealed that circACVR2A was a possible target of miR-1290. That means, miR-1290 can effectively suppress circACVR2A luciferase activity (*p* < 0.001; Figure 5(d)). Furthermore, we performed biotin-miR-1290 pulldown assay to check the interaction. Result showed biotin-miR-1290 can pull down more circACVR2A than biotin-NC (*p* < 0.001; Figure 5(e)). These data showed that circACVR2A would be possible target of miR-1290.

### 3.5. miR-1290 Inhibitor Alleviated the Effect of Downregulated circACVR2A on HGC-27 Proliferation and Metastasis In Vitro

**Figure 6:**
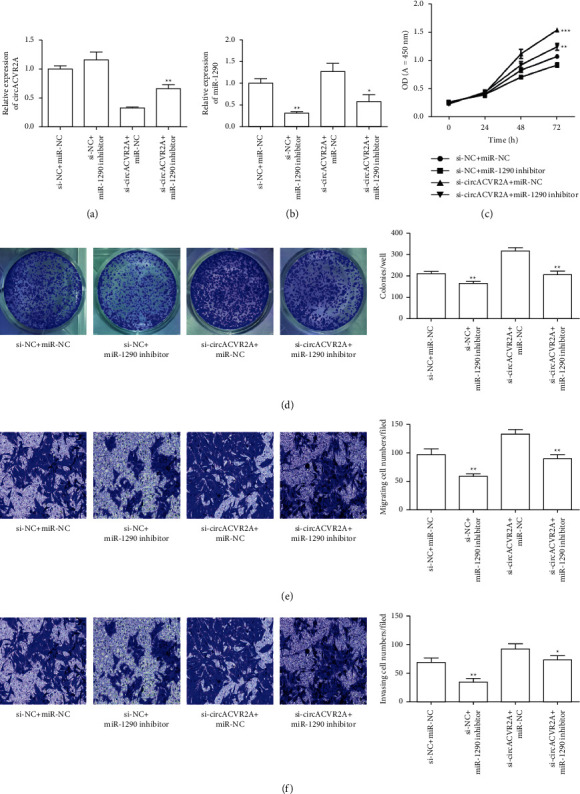
The downregulation of circACVR2A and miR-1290 on HGC-27 cell progression. (a) and (b) The relative level of circACVR2A and miR-1290 in HGC-27 cells transfecting with si-circACVR2A and miR-1290 inhibitor examined by qRT-PCR. The downregulation of circACVR2A and miR-1290 on HGC-27 cell proliferation (c), colony formation (d), migration (e), and invasion (f) was examined. The data is exhibited as the mean ± SD. ^*∗*^*p* < 0.05, ^*∗∗*^*p* < 0.01, and ^*∗∗∗*^*p* < 0.001.

**Figure 7:**
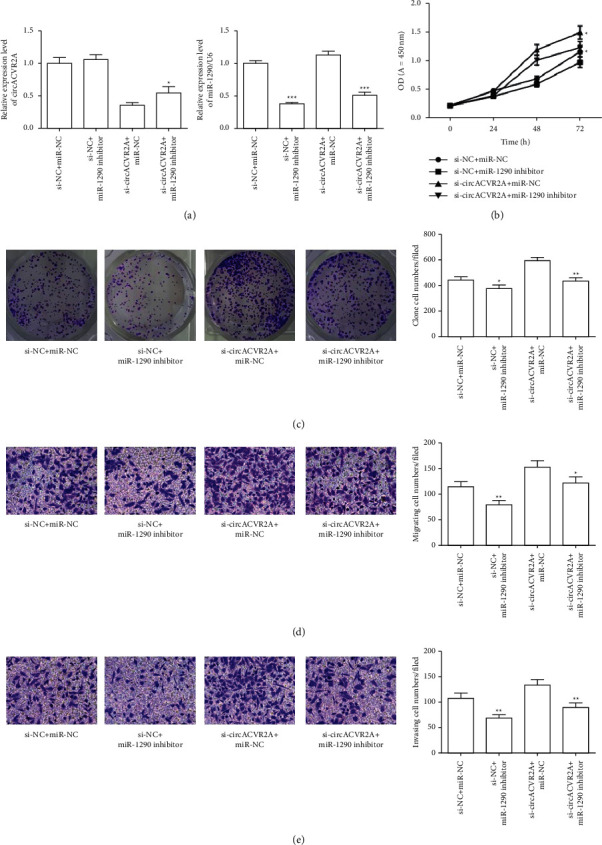
The downregulation of circACVR2A and miR-1290 on MKN-45 cell progression. (a) and (b) The relative level in MKN-45 cells examined by qRT-PCR. The downregulation of circACVR2A and miR-1290 on MKN-45 cell proliferation (c), colony formation (d), migration (e), and invasion (f) was examined. The data is exhibited as the mean ± SD. ^*∗*^*p* < 0.05, ^*∗∗*^*p* < 0.01, and ^*∗∗∗*^*p* < 0.001.

**Figure 8:**
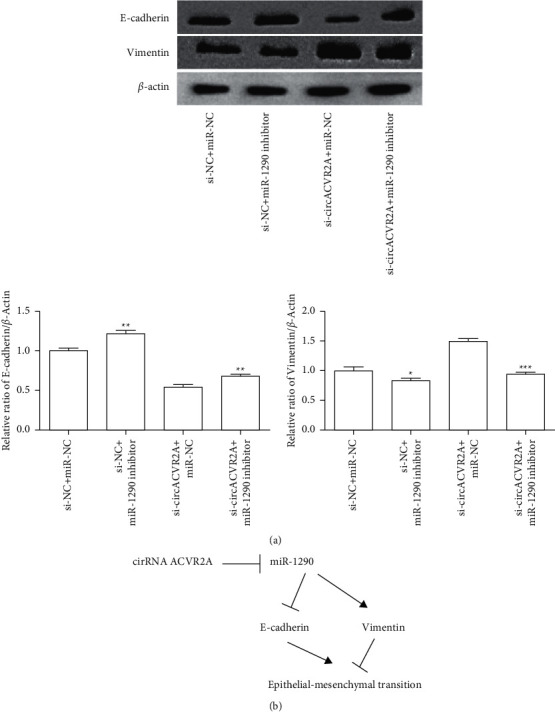
The downregulation of circACVR2A and miR-1290 on EMT pathway. (a) The expression of E-cadherin and Vimentin in HGC-27 cell transfected with si-circACVR2A and miR-1290 inhibitor was examined. (b) The graphical representation. The data is exhibited as the mean ± SD. ^*∗*^*p* < 0.05, ^*∗∗*^*p* < 0.01, and ^*∗∗∗*^*p* < 0.001.

To explore whether circACVR2A is affected via interacting with miR-1290, we examined whether miR-1290 inhibitor influenced circACVR2A expression in HGC-27. The data revealed that downregulated circACVR2A and miR-1290 significantly inhibited the level of circACVR2A (*p* < 0.01; Figure 6(a)), which also suppressed the miR-1290 expression (*p* < 0.05; Figure 6(b)) as compared with downregulation of circACVR2A. However, downregulated miR-1290 had no effect on the expression of circACVR2A. Downregulation of circACVR2A and miR-1290 inhibited proliferation and metastasis promoted by si-circACVR2A in HGC-27 (Figures 6(c)–6(f)). Obviously, these data revealed that circACVR2A and miR-1290 influenced the biological function on HGC-27 cells. miR-1290 had a reversed influence on GC cells against circACVR2A effect. To confirm the effect of circACVR2A through miR-1290, we performed experiments in MKN-45 cells. Results revealed the same expression pattern, cell proliferation, and metastasis ability in MKN-45 cells as HGC-27 (Figure 7). In addition, we tested the EMT-related protein expression. The downregulated miR-1290 remarkably enhanced E-cadherin expression and suppressed Vimentin expression, whereas downregulated circACVR2A obviously reversed the influence of these two proteins (Figure 8(a)).

## 4. Discussion

GC is one of the most common malignant cancers, and its incidence rate is increasing every year. Early-stage gastric cancer can be eradicated by surgery; however, most patients with gastric cancer represent no obvious clinical symptoms in early stage. When gastric cancer is found, the patient has entered into the middle or late stage, and the survival rate is low [6]. Therefore, earlier prognosis and therapy is emerging and necessary in early detection and treatment of gastric cancer. It is of great significance to find potential biomarkers for exploring the pathogenesis of gastric cancer.

circRNA was first found in RNA viruses and considered as the error splicing product in exon transcription [22–25]. Therefore, there is little attention in the exploitation of their value. However, with the application of high-throughput sequencing and biological analysis technologies, the potential function of circRNAs has suddenly been explored and become a research hotspot in biomedical field [20,26]. circRNA has high abundance and stability, with conservation of evolutionary species and tissue specificity. It widely exists in different types of tissues and could modulate the gene expression [21,27,28]. Due to their unique characteristics, circRNAs play a crucial function in cancer growth, metastasis, recurrence, and therapy resistance [29]. The circRNA-miRNA-mRNA axis exerts the function in carcinogenesis and has been proved to be a potential tumor diagnostic marker and therapeutic target [15,30]. Recently, a variety of circRNAs have been reported to be abnormally expressed in GC tissue or cell lines. Therefore, exploring GC related circRNAs as biomarkers or targets provides new insights into GC pathogenesis and brings novel possibilities for early diagnosis, prognosis, and effective treatment of GC [31]. In the present study, we showed that circACVR2A was significantly upregulated in GC, including tissues and cell lines. A series of functional experiments have shown that the downregulation of circACVR2A significantly enhanced the ability of proliferation and metastasis, while the upregulation of circACVR2A has the opposite effect on GC cells. Altogether, these findings revealed that circACVR2A functioned as a tumor suppressor gene.

circRNA usually has been utilized as miRNA sponge to regulate its downstream target genes and thus modulates biological effects [13,32]. Accumulating evidence has reported that a number of miRNAs could bind to circRNAs in human cancer [33,34]. In this study, we confirmed that circACVR2A interacted with miR-1290 in HGC-27 by the double luciferase reporter assay. Subsequently, the biological function of circACVR2A knockdown was counteracted by mir-1290. Accumulating evidence exhibited that abnormal miR-1290 expression has been causatively related to tumor progression. Similar data has been verified in other cancer cells, where downregulated miR-1290 may result in reducing growth, migration, and invasion in glioblastoma cells and lung cancer cells [35,36]. Therefore, the data demonstrated that the binding efficiency of circACVR2A and miR-1290 remarkably suppressed the antitumor capability of miR-1290.

To investigate the molecular basis for the involvement of circACVR2A in GC cells, we further examine whether circACVR2A influences the downstream pathways. In this study, our data indicated that circACVR2A could downregulate miR-1290 level via targeting its 3′-UTR to suppress the metastasis, strongly associated with epithelial-mesenchymal transition (EMT) pathway. EMT is a critical pathway for the metastatic dissemination. Therefore, repressing EMT signaling pathway is an effective road to suppress tumor formation and metastatic spread [37]. Hence, we evaluated the EMT-related factors and found that the downregulation of circACVR2A significantly inhibited E-cadherin level and remarkably enhanced Vimentin level. Obviously, circACVR2A is a regulator of the EMT signaling pathway in GC cells, but its regulatory role needs further investigated.

Taken together, we identified that circACVR2A was significantly upregulated in GC and represented it as a promising GC biomarker (Figure 8(b)). In addition, we for the first time demonstrated circACVR2A is exerted as a tumor suppressor in GC metastasis. This process is regulated by circACVR2A-mediated EMT pathway, bringing a new mechanism in the GC progression. It is suggested that circACVR2A may be a possible predictor and target for GC prognosis and treatment.

## Data Availability

All data are included within the article.
